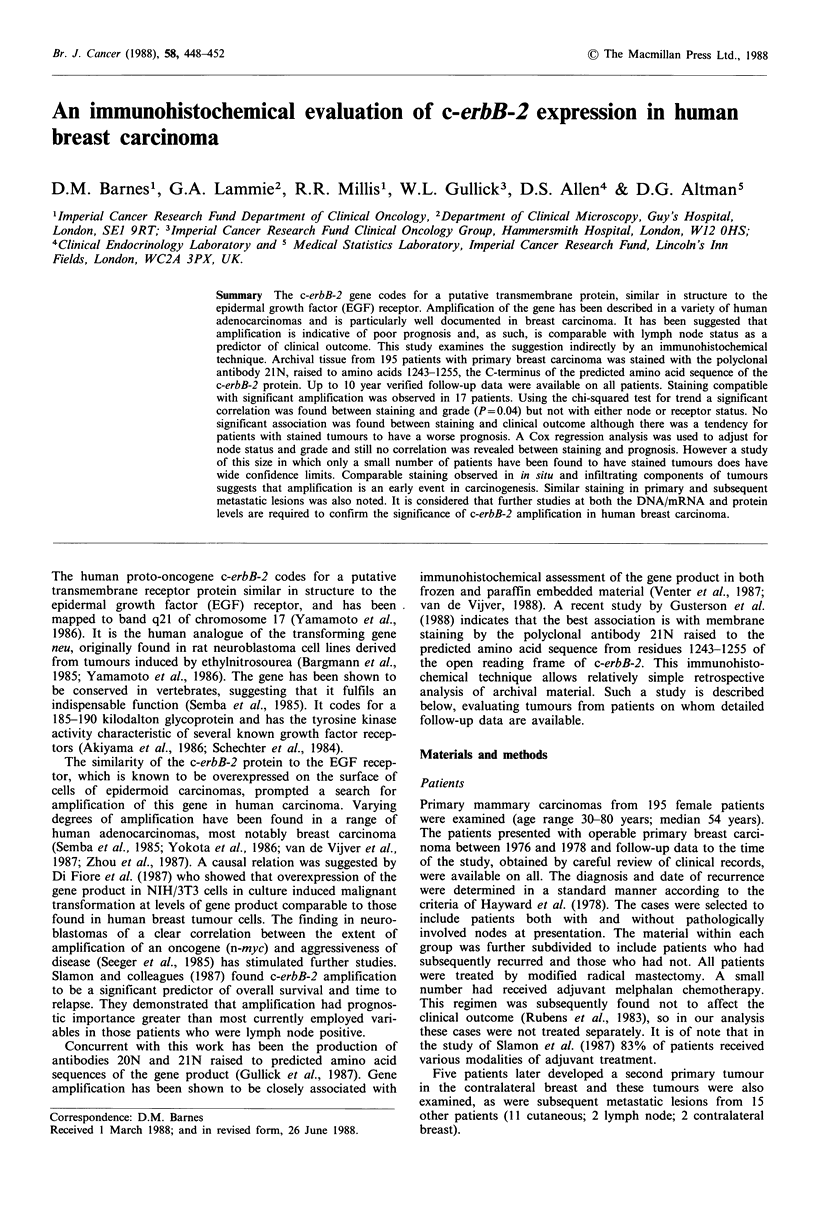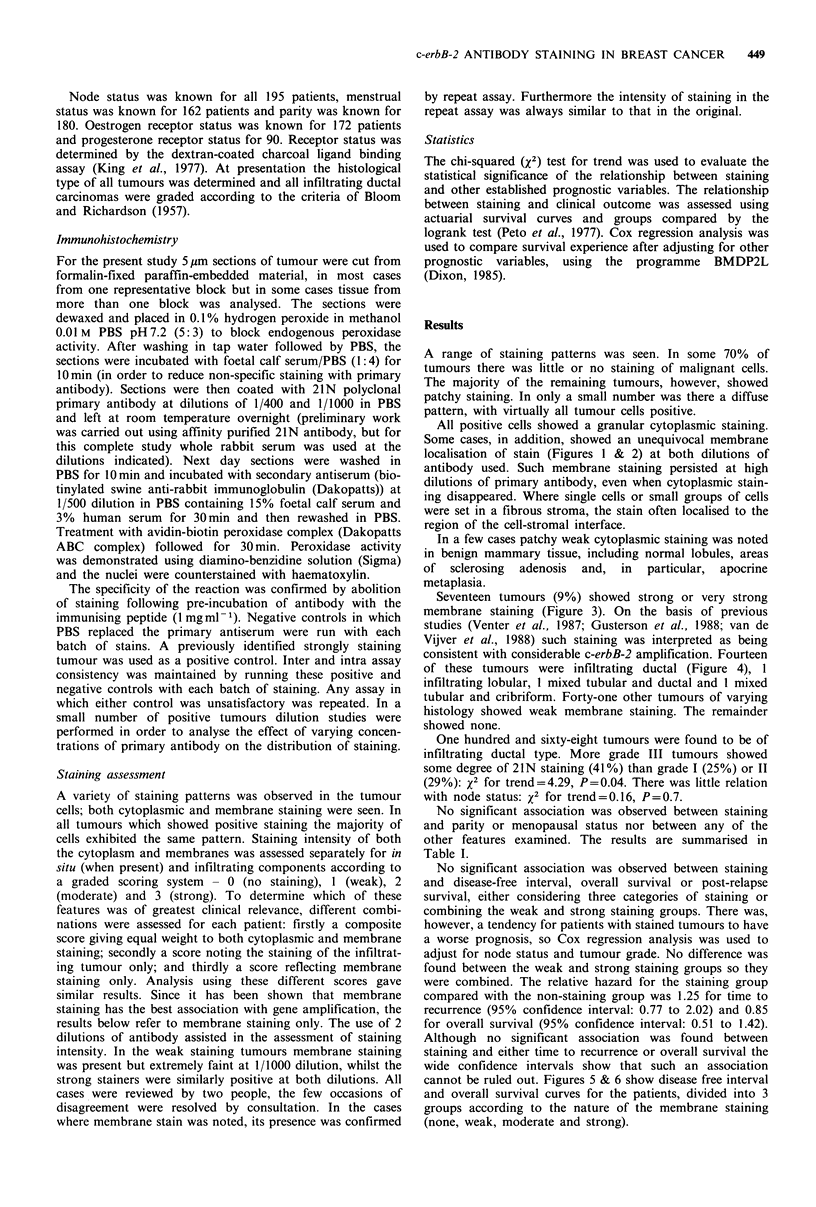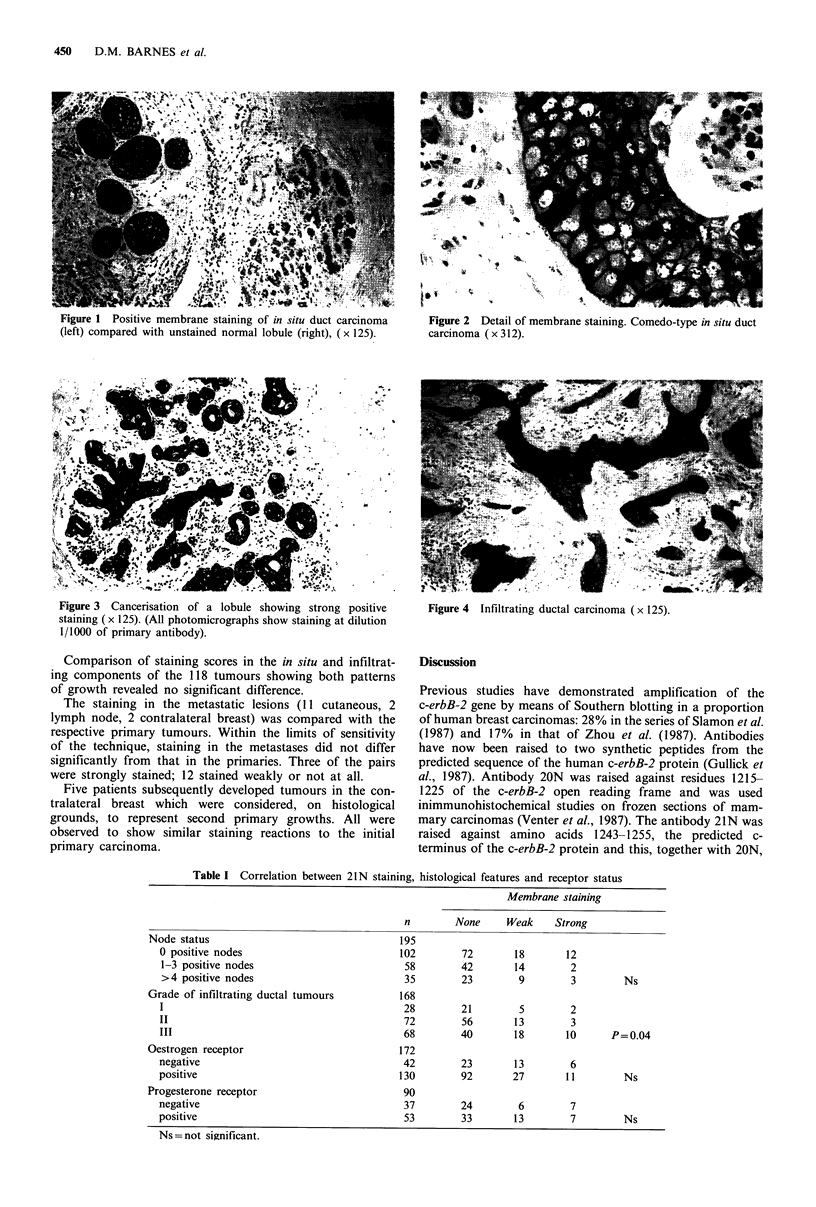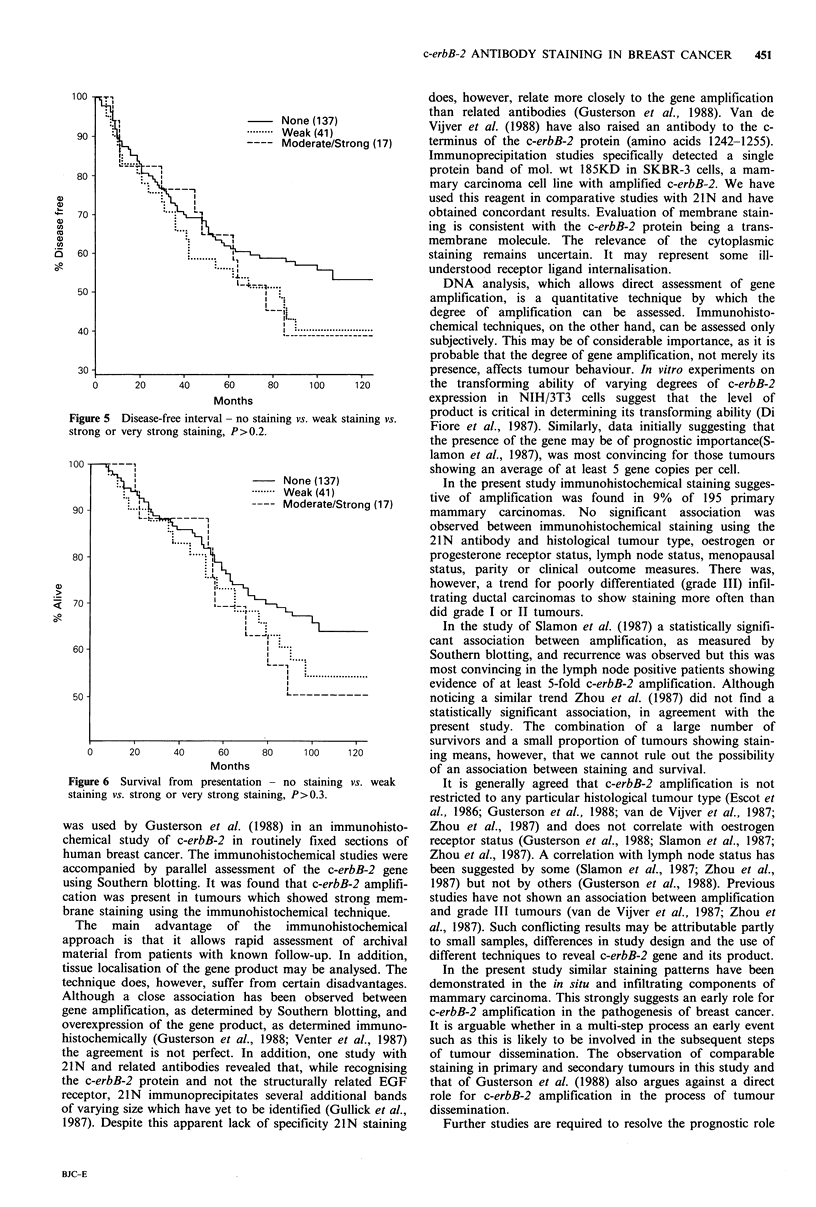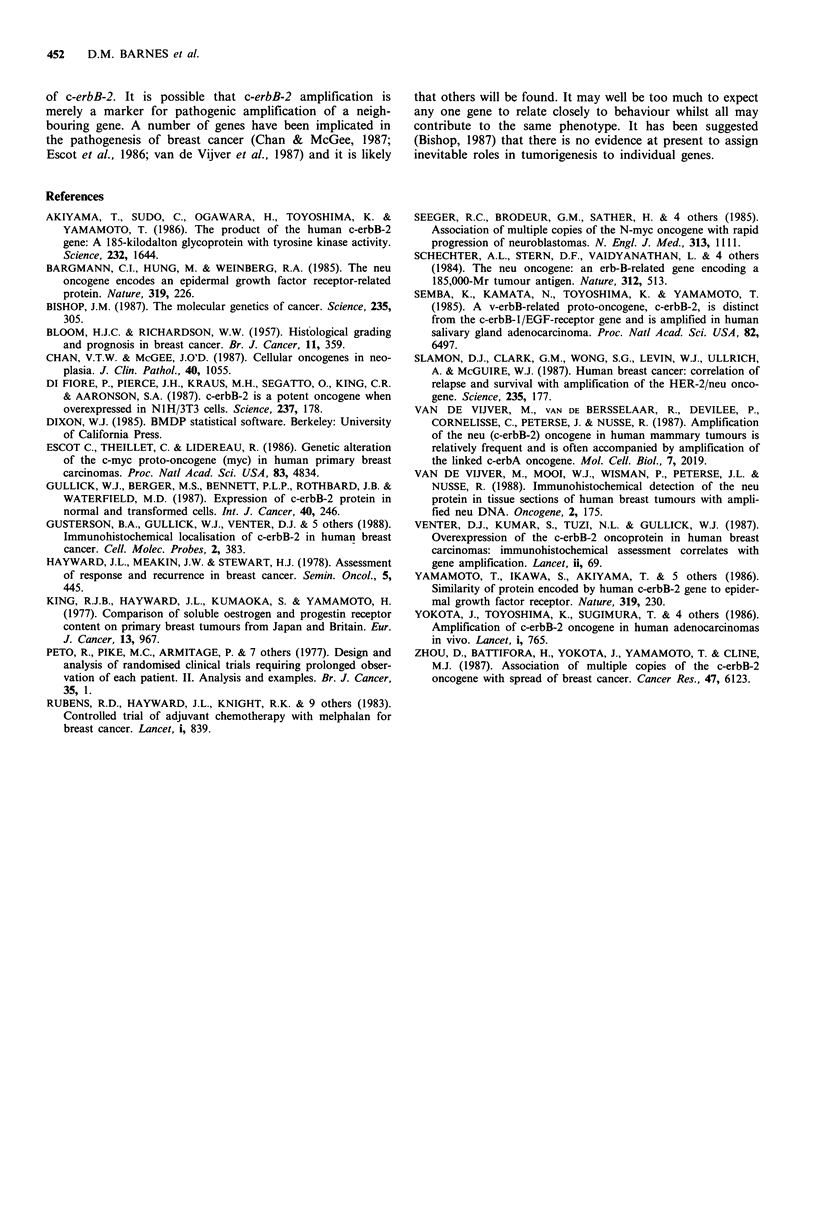# An immunohistochemical evaluation of c-erbB-2 expression in human breast carcinoma.

**DOI:** 10.1038/bjc.1988.238

**Published:** 1988-10

**Authors:** D. M. Barnes, G. A. Lammie, R. R. Millis, W. L. Gullick, D. S. Allen, D. G. Altman

**Affiliations:** Imperial Cancer Research Fund Department of Clinical Oncology, Guy's Hospital, London, UK.

## Abstract

**Images:**


*Br. J. Cancer* (1988), **58**, 448–452 © The Macmillan Press Ltd., 1988

### An immunohistochemical evaluation of c-*erbB-2* expression in human breast carcinoma

D.M. Barnes[1], G.A. Lammie[2], R.R. Millis[1], W.L. Gullick[3], D.S. Allen[4] & D.G. Altman[5]

[1]*Imperial Cancer Research Fund Department of Clinical Oncology,* [2]*Department of Clinical Microscopy, Guy's Hospital, London, SE1 9RT;* [3]*Imperial Cancer Research Fund Clinical Oncology Group, Hammersmith Hospital, London, W12 0HS;* [4]*Clinical Endocrinology Laboratory and* [5] *Medical Statistics Laboratory, Imperial Cancer Research Fund, Lincoln's Inn Fields, London, WC2A 3PX, UK.*

**Summary** The c-*erbB-2* gene codes for a putative transmembrane protein, similar in structure to the epidermal growth factor (EGF) receptor. Amplification of the gene has been described in a variety of human adenocarcinomas and is particularly well documented in breast carcinoma. It has been suggested that amplification is indicative of poor prognosis and, as such, is comparable with lymph node status as a predictor of clinical outcome. This study examines the suggestion indirectly by an immunohistochemical technique. Archival tissue from 195 patients with primary breast carcinoma was stained with the polyclonal antibody 21N, raised to amino acids 1243–1255, the C-terminus of the predicted amino acid sequence of the c-*erbB-2* protein. Up to 10 year verified follow-up data were available on all patients. Staining compatible with significant amplification was observed in 17 patients. Using the chi-squared test for trend a significant correlation was found between staining and grade ($P = 0.04$) but not with either node or receptor status. No significant association was found between staining and clinical outcome although there was a tendency for patients with stained tumours to have a worse prognosis. A Cox regression analysis was used to adjust for node status and grade and still no correlation was revealed between staining and prognosis. However a study of this size in which only a small number of patients have been found to have stained tumours does have wide confidence limits. Comparable staining observed in *in situ* and infiltrating components of tumours suggests that amplification is an early event in carcinogenesis. Similar staining in primary and subsequent metastatic lesions was also noted. It is considered that further studies at both the DNA/mRNA and protein levels are required to confirm the significance of c-*erbB-2* amplification in human breast carcinoma.

The human proto-oncogene c-*erbB-2* codes for a putative transmembrane receptor protein similar in structure to the epidermal growth factor (EGF) receptor, and has been mapped to band q21 of chromosome 17 (Yamamoto *et al.*, 1986). It is the human analogue of the transforming gene *neu*, originally found in rat neuroblastoma cell lines derived from tumours induced by ethylnitrosourea (Bargmann *et al.*, 1985; Yamamoto *et al.*, 1986). The gene has been shown to be conserved in vertebrates, suggesting that it fulfils an indispensable function (Semba *et al.*, 1985). It codes for a 185–190 kilodalton glycoprotein and has the tyrosine kinase activity characteristic of several known growth factor receptors (Akiyama *et al.*, 1986; Schechter *et al.*, 1984).

The similarity of the c-*erbB-2* protein to the EGF receptor, which is known to be overexpressed on the surface of cells of epidermoid carcinomas, prompted a search for amplification of this gene in human carcinoma. Varying degrees of amplification have been found in a range of human adenocarcinomas, most notably breast carcinoma (Semba *et al.*, 1985; Yokota *et al.*, 1986; van de Vijver *et al.*, 1987; Zhou *et al.*, 1987). A causal relation was suggested by Di Fiore *et al.* (1987) who showed that overexpression of the gene product in NIH/3T3 cells in culture induced malignant transformation at levels of gene product comparable to those found in human breast tumour cells. The finding in neuroblastomas of a clear correlation between the extent of amplification of an oncogene (n-*myc*) and aggressiveness of disease (Seeger *et al.*, 1985) has stimulated further studies. Slamon and colleagues (1987) found c-*erbB-2* amplification to be a significant predictor of overall survival and time to relapse. They demonstrated that amplification had prognostic importance greater than most currently employed variables in those patients who were lymph node positive.

Concurrent with this work has been the production of antibodies 20N and 21N raised to predicted amino acid sequences of the gene product (Gullick *et al.*, 1987). Gene amplification has been shown to be closely associated with immunohistochemical assessment of the gene product in both frozen and paraffin embedded material (Venter *et al.*, 1987; van de Vijver, 1988). A recent study by Gusterson *et al.* (1988) indicates that the best association is with membrane staining by the polyclonal antibody 21N raised to the predicted amino acid sequence from residues 1243–1255 of the open reading frame of c-*erbB-2*. This immunohistochemical technique allows relatively simple retrospective analysis of archival material. Such a study is described below, evaluating tumours from patients on whom detailed follow-up data are available.

Correspondence: D.M. Barnes
Received 1 March 1988; and in revised form, 26 June 1988.

### Materials and methods

### *Patients*

Primary mammary carcinomas from 195 female patients were examined (age range 30–80 years; median 54 years). The patients presented with operable primary breast carcinoma between 1976 and 1978 and follow-up data to the time of the study, obtained by careful review of clinical records, were available on all. The diagnosis and date of recurrence were determined in a standard manner according to the criteria of Hayward *et al.* (1978). The cases were selected to include patients both with and without pathologically involved nodes at presentation. The material within each group was further subdivided to include patients who had subsequently recurred and those who had not. All patients were treated by modified radical mastectomy. A small number had received adjuvant melphalan chemotherapy. This regimen was subsequently found not to affect the clinical outcome (Rubens *et al.*, 1983), so in our analysis these cases were not treated separately. It is of note that in the study of Slamon *et al.* (1987) 83% of patients received various modalities of adjuvant treatment.

Five patients later developed a second primary tumour in the contralateral breast and these tumours were also examined, as were subsequent metastatic lesions from 15 other patients (11 cutaneous; 2 lymph node; 2 contralateral breast).

Node status was known for all 195 patients, menstrual status was known for 162 patients and parity was known for 180. Oestrogen receptor status was known for 172 patients and progesterone receptor status for 90. Receptor status was determined by the dextran-coated charcoal ligand binding assay (King *et al.*, 1977). At presentation the histological type of all tumours was determined and all infiltrating ductal carcinomas were graded according to the criteria of Bloom and Richardson (1957).

*Immunohistochemistry*

For the present study 5 $\mu$m sections of tumour were cut from formalin-fixed paraffin-embedded material, in most cases from one representative block but in some cases tissue from more than one block was analysed. The sections were dewaxed and placed in 0.1% hydrogen peroxide in methanol 0.01 M PBS pH 7.2 (5:3) to block endogenous peroxidase activity. After washing in tap water followed by PBS, the sections were incubated with foetal calf serum/PBS (1:4) for 10 min (in order to reduce non-specific staining with primary antibody). Sections were then coated with 21N polyclonal primary antibody at dilutions of 1/400 and 1/1000 in PBS and left at room temperature overnight (preliminary work was carried out using affinity purified 21N antibody, but for this complete study whole rabbit serum was used at the dilutions indicated). Next day sections were washed in PBS for 10 min and incubated with secondary antiserum (biotinylated swine anti-rabbit immunoglobulin (Dakopatts)) at 1/500 dilution in PBS containing 15% foetal calf serum and 3% human serum for 30 min and then rewashed in PBS. Treatment with avidin-biotin peroxidase complex (Dakopatts ABC complex) followed for 30 min. Peroxidase activity was demonstrated using diamino-benzidine solution (Sigma) and the nuclei were counterstained with haematoxylin.

The specificity of the reaction was confirmed by abolition of staining following pre-incubation of antibody with the immunising peptide (1 mg ml$^{-1}$). Negative controls in which PBS replaced the primary antiserum were run with each batch of stains. A previously identified strongly staining tumour was used as a positive control. Inter and intra assay consistency was maintained by running these positive and negative controls with each batch of staining. Any assay in which either control was unsatisfactory was repeated. In a small number of positive tumours dilution studies were performed in order to analyse the effect of varying concentrations of primary antibody on the distribution of staining.

*Staining assessment*

A variety of staining patterns was observed in the tumour cells; both cytoplasmic and membrane staining were seen. In all tumours which showed positive staining the majority of cells exhibited the same pattern. Staining intensity of both the cytoplasm and membranes was assessed separately for *in situ* (when present) and infiltrating components according to a graded scoring system – 0 (no staining), 1 (weak), 2 (moderate) and 3 (strong). To determine which of these features was of greatest clinical relevance, different combinations were assessed for each patient: firstly a composite score giving equal weight to both cytoplasmic and membrane staining; secondly a score noting the staining of the infiltrating tumour only; and thirdly a score reflecting membrane staining only. Analysis using these different scores gave similar results. Since it has been shown that membrane staining has the best association with gene amplification, the results below refer to membrane staining only. The use of 2 dilutions of antibody assisted in the assessment of staining intensity. In the weak staining tumours membrane staining was present but extremely faint at 1/1000 dilution, whilst the strong stainers were similarly positive at both dilutions. All cases were reviewed by two people, the few occasions of disagreement were resolved by consultation. In the cases where membrane stain was noted, its presence was confirmed by repeat assay. Furthermore the intensity of staining in the repeat assay was always similar to that in the original.

*Statistics*

The chi-squared ($\chi^2$) test for trend was used to evaluate the statistical significance of the relationship between staining and other established prognostic variables. The relationship between staining and clinical outcome was assessed using actuarial survival curves and groups compared by the logrank test (Peto *et al.*, 1977). Cox regression analysis was used to compare survival experience after adjusting for other prognostic variables, using the programme BMDP2L (Dixon, 1985).

### Results

A range of staining patterns was seen. In some 70% of tumours there was little or no staining of malignant cells. The majority of the remaining tumours, however, showed patchy staining. In only a small number was there a diffuse pattern, with virtually all tumour cells positive.

All positive cells showed a granular cytoplasmic staining. Some cases, in addition, showed an unequivocal membrane localisation of stain (Figures 1 & 2) at both dilutions of antibody used. Such membrane staining persisted at high dilutions of primary antibody, even when cytoplasmic staining disappeared. Where single cells or small groups of cells were set in a fibrous stroma, the stain often localised to the region of the cell-stromal interface.

In a few cases patchy weak cytoplasmic staining was noted in benign mammary tissue, including normal lobules, areas of sclerosing adenosis and, in particular, apocrine metaplasia.

Seventeen tumours (9%) showed strong or very strong membrane staining (Figure 3). On the basis of previous studies (Venter *et al.*, 1987; Gusterson *et al.*, 1988; van de Vijver *et al.*, 1988) such staining was interpreted as being consistent with considerable c-*erbB-2* amplification. Fourteen of these tumours were infiltrating ductal (Figure 4), 1 infiltrating lobular, 1 mixed tubular and ductal and 1 mixed tubular and cribriform. Forty-one other tumours of varying histology showed weak membrane staining. The remainder showed none.

One hundred and sixty-eight tumours were found to be of infiltrating ductal type. More grade III tumours showed some degree of 21N staining (41%) than grade I (25%) or II (29%): $\chi^2$ for trend = 4.29, $P = 0.04$. There was little relation with node status: $\chi^2$ for trend = 0.16, $P = 0.7$.

No significant association was observed between staining and parity or menopausal status nor between any of the other features examined. The results are summarised in Table I.

No significant association was observed between staining and disease-free interval, overall survival or post-relapse survival, either considering three categories of staining or combining the weak and strong staining groups. There was, however, a tendency for patients with stained tumours to have a worse prognosis, so Cox regression analysis was used to adjust for node status and tumour grade. No difference was found between the weak and strong staining groups so they were combined. The relative hazard for the staining group compared with the non-staining group was 1.25 for time to recurrence (95% confidence interval: 0.77 to 2.02) and 0.85 for overall survival (95% confidence interval: 0.51 to 1.42). Although no significant association was found between staining and either time to recurrence or overall survival the wide confidence intervals show that such an association cannot be ruled out. Figures 5 & 6 show disease free interval and overall survival curves for the patients, divided into 3 groups according to the nature of the membrane staining (none, weak, moderate and strong).

**Figure 1** Positive membrane staining of *in situ* duct carcinoma (left) compared with unstained normal lobule (right), (×125).

**Figure 2** Detail of membrane staining. Comedo-type *in situ* duct carcinoma (×312).

**Figure 3** Cancerisation of a lobule showing strong positive staining (×125). (All photomicrographs show staining at dilution 1/1000 of primary antibody).

**Figure 4** Infiltrating ductal carcinoma (×125).

Comparison of staining scores in the *in situ* and infiltrating components of the 118 tumours showing both patterns of growth revealed no significant difference.

The staining in the metastatic lesions (11 cutaneous, 2 lymph node, 2 contralateral breast) was compared with the respective primary tumours. Within the limits of sensitivity of the technique, staining in the metastases did not differ significantly from that in the primaries. Three of the pairs were strongly stained; 12 stained weakly or not at all.

Five patients subsequently developed tumours in the contralateral breast which were considered, on histological grounds, to represent second primary growths. All were observed to show similar staining reactions to the initial primary carcinoma.

### Discussion

Previous studies have demonstrated amplification of the c-*erbB-2* gene by means of Southern blotting in a proportion of human breast carcinomas: 28% in the series of Slamon *et al.* (1987) and 17% in that of Zhou *et al.* (1987). Antibodies have now been raised to two synthetic peptides from the predicted sequence of the human c-*erbB-2* protein (Gullick *et al.*, 1987). Antibody 20N was raised against residues 1215–1225 of the c-*erbB-2* open reading frame and was used inimmunohistochemical studies on frozen sections of mammary carcinomas (Venter *et al.*, 1987). The antibody 21N was raised against amino acids 1243–1255, the predicted c-terminus of the c-*erbB-2* protein and this, together with 20N,

**Table I** Correlation between 21N staining, histological features and receptor status

| | | Membrane staining | | | |
|---|---|---|---|---|---|
| | *n* | *None* | *Weak* | *Strong* | |
| Node status | 195 | | | | |
| 0 positive nodes | 102 | 72 | 18 | 12 | |
| 1–3 positive nodes | 58 | 42 | 14 | 2 | |
| >4 positive nodes | 35 | 23 | 9 | 3 | Ns |
| Grade of infiltrating ductal tumours | 168 | | | | |
| I | 28 | 21 | 5 | 2 | |
| II | 72 | 56 | 13 | 3 | |
| III | 68 | 40 | 18 | 10 | $P=0.04$ |
| Oestrogen receptor | 172 | | | | |
| negative | 42 | 23 | 13 | 6 | |
| positive | 130 | 92 | 27 | 11 | Ns |
| Progesterone receptor | 90 | | | | |
| negative | 37 | 24 | 6 | 7 | |
| positive | 53 | 33 | 13 | 7 | Ns |

Ns = not significant.

**Figure 5** Disease-free interval – no staining *vs.* weak staining *vs.* strong or very strong staining, $P > 0.2$.

**Figure 6** Survival from presentation – no staining *vs.* weak staining *vs.* strong or very strong staining, $P > 0.3$.

was used by Gusterson *et al.* (1988) in an immunohistochemical study of c-*erbB-2* in routinely fixed sections of human breast cancer. The immunohistochemical studies were accompanied by parallel assessment of the c-*erbB-2* gene using Southern blotting. It was found that c-*erbB-2* amplification was present in tumours which showed strong membrane staining using the immunohistochemical technique.

The main advantage of the immunohistochemical approach is that it allows rapid assessment of archival material from patients with known follow-up. In addition, tissue localisation of the gene product may be analysed. The technique does, however, suffer from certain disadvantages. Although a close association has been observed between gene amplification, as determined by Southern blotting, and overexpression of the gene product, as determined immunohistochemically (Gusterson *et al.*, 1988; Venter *et al.*, 1987) the agreement is not perfect. In addition, one study with 21N and related antibodies revealed that, while recognising the c-*erbB-2* protein and not the structurally related EGF receptor, 21N immunoprecipitates several additional bands of varying size which have yet to be identified (Gullick *et al.*, 1987). Despite this apparent lack of specificity 21N staining does, however, relate more closely to the gene amplification than related antibodies (Gusterson *et al.*, 1988). Van de Vijver *et al.* (1988) have also raised an antibody to the c-terminus of the c-*erbB-2* protein (amino acids 1242–1255). Immunoprecipitation studies specifically detected a single protein band of mol. wt 185KD in SKBR-3 cells, a mammary carcinoma cell line with amplified c-*erbB-2*. We have used this reagent in comparative studies with 21N and have obtained concordant results. Evaluation of membrane staining is consistent with the c-*erbB-2* protein being a transmembrane molecule. The relevance of the cytoplasmic staining remains uncertain. It may represent some ill-understood receptor ligand internalisation.

DNA analysis, which allows direct assessment of gene amplification, is a quantitative technique by which the degree of amplification can be assessed. Immunohistochemical techniques, on the other hand, can be assessed only subjectively. This may be of considerable importance, as it is probable that the degree of gene amplification, not merely its presence, affects tumour behaviour. *In vitro* experiments on the transforming ability of varying degrees of c-*erbB-2* expression in NIH/3T3 cells suggest that the level of product is critical in determining its transforming ability (Di Fiore *et al.*, 1987). Similarly, data initially suggesting that the presence of the gene may be of prognostic importance(Slamon *et al.*, 1987), was most convincing for those tumours showing an average of at least 5 gene copies per cell.

In the present study immunohistochemical staining suggestive of amplification was found in 9% of 195 primary mammary carcinomas. No significant association was observed between immunohistochemical staining using the 21N antibody and histological tumour type, oestrogen or progesterone receptor status, lymph node status, menopausal status, parity or clinical outcome measures. There was, however, a trend for poorly differentiated (grade III) infiltrating ductal carcinomas to show staining more often than did grade I or II tumours.

In the study of Slamon *et al.* (1987) a statistically significant association between amplification, as measured by Southern blotting, and recurrence was observed but this was most convincing in the lymph node positive patients showing evidence of at least 5-fold c-*erbB-2* amplification. Although noticing a similar trend Zhou *et al.* (1987) did not find a statistically significant association, in agreement with the present study. The combination of a large number of survivors and a small proportion of tumours showing staining means, however, that we cannot rule out the possibility of an association between staining and survival.

It is generally agreed that c-*erbB-2* amplification is not restricted to any particular histological tumour type (Escot *et al.*, 1986; Gusterson *et al.*, 1988; van de Vijver *et al.*, 1987; Zhou *et al.*, 1987) and does not correlate with oestrogen receptor status (Gusterson *et al.*, 1988; Slamon *et al.*, 1987; Zhou *et al.*, 1987). A correlation with lymph node status has been suggested by some (Slamon *et al.*, 1987; Zhou *et al.*, 1987) but not by others (Gusterson *et al.*, 1988). Previous studies have not shown an association between amplification and grade III tumours (van de Vijver *et al.*, 1987; Zhou *et al.*, 1987). Such conflicting results may be attributable partly to small samples, differences in study design and the use of different techniques to reveal c-*erbB-2* gene and its product.

In the present study similar staining patterns have been demonstrated in the *in situ* and infiltrating components of mammary carcinoma. This strongly suggests an early role for c-*erbB-2* amplification in the pathogenesis of breast cancer. It is arguable whether in a multi-step process an early event such as this is likely to be involved in the subsequent steps of tumour dissemination. The observation of comparable staining in primary and secondary tumours in this study and that of Gusterson *et al.* (1988) also argues against a direct role for c-*erbB-2* amplification in the process of tumour dissemination.

Further studies are required to resolve the prognostic role of c-*erbB-2*. It is possible that c-*erbB-2* amplification is merely a marker for pathogenic amplification of a neighbouring gene. A number of genes have been implicated in the pathogenesis of breast cancer (Chan & McGee, 1987; Escot *et al.*, 1986; van de Vijver *et al.*, 1987) and it is likely that others will be found. It may well be too much to expect any one gene to relate closely to behaviour whilst all may contribute to the same phenotype. It has been suggested (Bishop, 1987) that there is no evidence at present to assign inevitable roles in tumorigenesis to individual genes.

### References

AKIYAMA, T., SUDO, C., OGAWARA, H., TOYOSHIMA, K. & YAMAMOTO, T. (1986). The product of the human c-erbB-2 gene: A 185-kilodalton glycoprotein with tyrosine kinase activity. *Science*, **232**, 1644.

BARGMANN, C.I., HUNG, M. & WEINBERG, R.A. (1985). The neu oncogene encodes an epidermal growth factor receptor-related protein. *Nature*, **319**, 226.

BISHOP, J.M. (1987). The molecular genetics of cancer. *Science*, **235**, 305.

BLOOM, H.J.C. & RICHARDSON, W.W. (1957). Histological grading and prognosis in breast cancer. *Br. J. Cancer*, **11**, 359.

CHAN, V.T.W. & McGEE, J.O'D. (1987). Cellular oncogenes in neoplasia. *J. Clin. Pathol.*, **40**, 1055.

DI FIORE, P., PIERCE, J.H., KRAUS, M.H., SEGATTO, O., KING, C.R. & AARONSON, S.A. (1987). c-erbB-2 is a potent oncogene when overexpressed in N1H/3T3 cells. *Science*, **237**, 178.

DIXON, W.J. (1985). BMDP statistical software. Berkeley: University of California Press.

ESCOT C., THEILLET, C. & LIDEREAU, R. (1986). Genetic alteration of the c-myc proto-oncogene (myc) in human primary breast carcinomas. *Proc. Natl Acad. Sci. USA*, **83**, 4834.

GULLICK, W.J., BERGER, M.S., BENNETT, P.L.P., ROTHBARD, J.B. & WATERFIELD, M.D. (1987). Expression of c-erbB-2 protein in normal and transformed cells. *Int. J. Cancer*, **40**, 246.

GUSTERSON, B.A., GULLICK, W.J., VENTER, D.J. & 5 others (1988). Immunohistochemical localisation of c-erbB-2 in human breast cancer. *Cell. Molec. Probes*, **2**, 383.

HAYWARD, J.L., MEAKIN, J.W. & STEWART, H.J. (1978). Assessment of response and recurrence in breast cancer. *Semin. Oncol.*, **5**, 445.

KING, R.J.B., HAYWARD, J.L., KUMAOKA, S. & YAMAMOTO, H. (1977). Comparison of soluble oestrogen and progestin receptor content on primary breast tumours from Japan and Britain. *Eur. J. Cancer*, **13**, 967.

PETO, R., PIKE, M.C., ARMITAGE, P. & 7 others (1977). Design and analysis of randomised clinical trials requiring prolonged observation of each patient. II. Analysis and examples. *Br. J. Cancer*, **35**, 1.

RUBENS, R.D., HAYWARD, J.L., KNIGHT, R.K. & 9 others (1983). Controlled trial of adjuvant chemotherapy with melphalan for breast cancer. *Lancet*, **i**, 839.

SEEGER, R.C., BRODEUR, G.M., SATHER, H. & 4 others (1985). Association of multiple copies of the N-myc oncogene with rapid progression of neuroblastomas. *N. Engl. J. Med.*, **313**, 1111.

SCHECHTER, A.L., STERN, D.F., VAIDYANATHAN, L. & 4 others (1984). The neu oncogene: an erb-B-related gene encoding a 185,000-Mr tumour antigen. *Nature*, **312**, 513.

SEMBA, K., KAMATA, N., TOYOSHIMA, K. & YAMAMOTO, T. (1985). A v-erbB-related proto-oncogene, c-erbB-2, is distinct from the c-erbB-1/EGF-receptor gene and is amplified in human salivary gland adenocarcinoma. *Proc. Natl Acad. Sci. USA*, **82**, 6497.

SLAMON, D.J., CLARK, G.M., WONG, S.G., LEVIN, W.J., ULLRICH, A. & McGUIRE, W.J. (1987). Human breast cancer: correlation of relapse and survival with amplification of the HER-2/neu oncogene. *Science*, **235**, 177.

VAN DE VIJVER, M., VAN DE BERSSELAAR, R., DEVILEE, P., CORNELISSE, C., PETERSE, J. & NUSSE, R. (1987). Amplification of the neu (c-erbB-2) oncogene in human mammary tumours is relatively frequent and is often accompanied by amplification of the linked c-erbA oncogene. *Mol. Cell. Biol.*, **7**, 2019.

VAN DE VIJVER, M., MOOI, W.J., WISMAN, P., PETERSE, J.L. & NUSSE, R. (1988). Immunohistochemical detection of the neu protein in tissue sections of human breast tumours with amplified neu DNA. *Oncogene*, **2**, 175.

VENTER, D.J., KUMAR, S., TUZI, N.L. & GULLICK, W.J. (1987). Overexpression of the c-erbB-2 oncoprotein in human breast carcinomas: immunohistochemical assessment correlates with gene amplification. *Lancet*, **ii**, 69.

YAMAMOTO, T., IKAWA, S., AKIYAMA, T. & 5 others (1986). Similarity of protein encoded by human c-erbB-2 gene to epidermal growth factor receptor. *Nature*, **319**, 230.

YOKOTA, J., TOYOSHIMA, K., SUGIMURA, T. & 4 others (1986). Amplification of c-erbB-2 oncogene in human adenocarcinomas in vivo. *Lancet*, **i**, 765.

ZHOU, D., BATTIFORA, H., YOKOTA, J., YAMAMOTO, T. & CLINE, M.J. (1987). Association of multiple copies of the c-erbB-2 oncogene with spread of breast cancer. *Cancer Res.*, **47**, 6123.